# Association between Periodontal Disease and Obesity: Umbrella Review

**DOI:** 10.3390/medicina60040621

**Published:** 2024-04-11

**Authors:** Heber Isac Arbildo-Vega, Fredy Hugo Cruzado-Oliva, Franz Tito Coronel-Zubiate, Rubén Aguirre-Ipenza, Joan Manuel Meza-Málaga, Sara Antonieta Luján-Valencia, Eduardo Luján-Urviola, Carlos Alberto Farje-Gallardo

**Affiliations:** 1 Faculty of Dentistry, Dentistry School, San Martin de Porres University, Chiclayo 14012, Peru; hiav30@gmail.com; 2 Faculty of Human Medicine, Human Medicine School, San Martín de Porres University, Chiclayo 14012, Peru; 3 Faculty of Stomatology, Stomatology School, Nacional University of Trujillo, Trujillo 13001, Peru; fcruza-do@unitru.edu.pe; 4 Faculty of Health Sciences, Stomatology School, Toribio Rodríguez of Mendoza National University of Amazonas, Chachapoyas 01001, Peru; franz.coronel@untrm.edu.pe (F.T.C.-Z.); carlos.farje@untrm.edu.pe (C.A.F.-G.); 5 Faculty of Health Sciences, Continental University, Lima 15046, Peru; 6 Faculty of Dentistry, Dentistry School, Catholic University of Santa Maria, Arequipa 04013, Peru; jmezam@ucsm.edu.pe (J.M.M.-M.); slujan@ucsm.edu.pe (S.A.L.-V.); 7 Faculty of Medicine, Medicine School, Catholic University of Santa Maria, Arequipa 04013, Peru; 8 Postgraduate School, Catholic University of Santa Maria, Arequipa 04013, Peru; 9 Faculty of Dentistry, Néstor Cáceres Velásquez Andean University, Juliaca 21104, Peru; edulujan040@hotmail.com

**Keywords:** obesity, overweight, periodontal disease, periodontitis, review

## Abstract

*Objective*: Determine the association between periodontal disease (PD) and obesity through an umbrella review. *Materials and Methods*: A search for information until March 2024 was carried out in the following electronic databases: PubMed, Cochrane library, Scopus, SciELO, Web of Science, Google Scholar, Proquest Dissertations and Theses, and OpenGrey. We included studies that were systematic reviews (SR) with or without meta-analysis, without time or language restrictions, that evaluated primary studies that associated PD with obesity. Literary or narrative reviews, rapid reviews, intervention studies, observational studies, preclinical and basic research, summaries, comments, case reports, protocols, personal opinions, letters, and posters were excluded. The AMSTAR-2 tool was used to determine the quality and overall confidence of the included studies. *Results*: The preliminary search yielded a total of 419 articles, discarding those that did not meet the selection criteria, leaving only 14 articles. All studies reported that PD was associated with obesity, with an OR and RR ranging from 1.1 to 1.46 and 1.64 to 2.21, respectively. *Conclusions*: Based on the results and conclusions of the SR with a high overall confidence level, PD is associated with obesity.

## 1. Introduction

In recent years, evidence has accumulated on the relationships between oral diseases such as periodontitis and various systemic diseases, known as periodontal medicine [1]. The strongest associations, supported by a significant amount of evidence, include cardiovascular disease, adverse pregnancy outcomes, respiratory disease, and diabetes mellitus [2,3,4,5]. In 2013, the European Federation of Periodontology (EFP) and the American Academy of Periodontology (AAP) organized workshops focusing on these associations, especially cardiovascular disease, diabetes, and adverse pregnancy outcomes [6,7,8,9]. However, Linden et al. [10] explored lesser-known associations, such as chronic kidney disease, rheumatoid arthritis, cognitive decline, inflammatory cancers, and obesity. Although some modest associations were found between periodontitis and obesity, connections with other diseases are weaker and are subject to limitations in the definition of periodontal disease (PD) and the control of confounding factors in the studies [10]. A systematic mapping of clinical trial registries conducted in 2016 reported that 57 systemic conditions are currently being investigated for possible links to PDs [11].

Obesity and being overweight represent a significant public health challenge in the modern era [12], with prevalence steadily increasing globally since 1980 [13]. This problem affects about a third of the world’s population, with higher rates among men for overweight and among women for obesity [13,14]. Furthermore, each year, obesity and being overweight cause the death of around 3.4 million people [13,15,16]. The World Health Organization (WHO) defines an adult as overweight if the body mass index (BMI) is greater than or equal to 25 and obese if the BMI is greater than or equal to 30 [17], while, for children and adolescents, it defines that they will be overweight if the BMI is greater than or equal to the 85th percentile and obese if the BMI is greater than or equal to the 95th percentile [18].

Obesity is associated with an increased risk of serious diseases, such as heart disease, hypertension, type 2 diabetes, and several types of cancer, and contributes to increased medical costs [13,19,20]. Despite genetic predisposition, environmental changes, availability of high-fat foods, and decreased physical activity have contributed to rising obesity rates worldwide [21]. The BMI is a commonly used measure to assess the relative amount of body fat in a person [22,23,24] and has been associated with metabolism [25,26] and oral health [27]. Obesity is associated with dental problems such as dental caries, periodontitis, and tooth loss, and inflammation is thought to play a key role in this relationship [27,28].

Only one umbrella systematic review [1] on the associations of PD with obesity has been published in the scientific literature. However, a general synthesis and evaluation of all systematic reviews taken together, including those published in recent years, has not yet been performed. Therefore, the purpose of this umbrella review was to summarize the available evidence and answer the following specific question: “What do we know so far about the association of PD and obesity?” and what is the overall confidence of systematic reviews assessing this topic?

## 2. Materials and Methods

### 2.1. Protocol and Registration

A protocol was carried out based on the Preferred Reporting Items for Systematic Reviews and Meta-Analysis Protocols (PRISMA-P) [29] and registered in the Prospective Registry of Systematic Reviews (PROSPERO) [30]. The registry is publicly available under the number CRD42024521090. In addition, the report of this study is based on the Preferred Reporting Items for Overview of Systematic Reviews Checklist (PRIO-harms) [31]. Ethical approval was not required for this umbrella review.

The focused question was formulated using the PECO format (population, exposure, comparison, and outcomes), as detailed below:

− Population: people of all ages.
− Exposure: people with obesity (BMI ≥ 30 or ≥95th percentile) and/or overweight (BMI ≥ 25 or ≥85th percentile).
− Comparison: people with normal weight (BMI ≥ 18.5 or ≥5th percentile).
− Outcomes: association with periodontal disease.

### 2.2. Eligibility Criteria and Results of Interest

The included studies were systematic reviews (SR) with or without meta-analysis, without time and language restrictions, that evaluated primary studies that reported the association between PD and obesity. 

Literature or narrative reviews, rapid reviews, intervention studies, observational studies, preclinical and basic research, abstracts, commentaries, case reports, protocols, personal opinions, letters, and posters were excluded.

### 2.3. Sources of Information, Search Strategy, and Additional Search for Primary Studies

An electronic search was performed on 5 March 2024 in five databases (Pubmed, Cochrane database, Scielo, Web of Science, and Scopus). Gray literature was also consulted through Google Scholar, Proquest Dissertations and Theses, and OpenGrey. In addition, the reference lists of the included studies were reviewed. The found articles were exported to reference management software (Zotero^®^ 6.0, Center for History and New Media, Fairfax, VA, USA) and duplicate articles were removed. The search strategy adopted for each database is shown in Table 1.

### 2.4. Data Management and Selection Process

The identified articles were entered into Rayyan^®^ Online Software https://www.rayyan.ai/, accessed on 4 April 2024 (Qatar Research Institute of Computing, Doha, Qatar). The selection of the studies was performed in 2 phases; in phase 1, two reviewers (F.C.O. and F.C.Z.) independently selected the studies by reading the title and abstract; then, phase 2 was carried out, which consisted of reading the full text, performed independently by the same two reviewers. A third reviewer (H.A.) was consulted in case of disagreement.

### 2.5. Data Collection Process

Data from the studies were independently collected in duplicate using a table previously formulated by two reviewers (F.C.O. and R.A.). The data were cross-checked and disagreements resolved by the third review author (H.A.). The following information was extracted from the selected articles: authors, year of publication, study design, design of the primary studies included, number of studies included in the qualitative and quantitative analysis, results, main conclusions, mentions of what was used or carried out: PRISMA, PROSPERO, and Grading of Recommendations Assessment, Development and Assessment (GRADE), and meta-analysis.

### 2.6. Assessment of Methodological Quality, Quality of Evidence, and Meta-Bias

The evaluation of the methodological quality of the included SRs was performed independently in duplicate by two reviewers (J.M. and S.L.), calibrated (Kappa 0.85), using the AMSTAR-2 checklist (A MeaSurement Tool to Assess Systemic Reviews) [32]. The AMSTAR-2 evaluates the methodological quality of the SR through 16 questions that can be answered with three possible answers: “yes”, “no”, or “partially yes”. The overall confidence rating (high, moderate, low, and critically low) in the studies was assessed as suggested by Shea et al. [32].

### 2.7. Summary of Measures

In the case of an SR without meta-analysis, the results shown in odds ratio (OR), hazard ratio (HR), incidence risk ratio (IRR), or prevalence ratio (PR) in ranges or intervals were considered. If the SR presents meta-analysis, we consider the results that were shown with OR, risk/rate ratio (RR), or standardized mean difference (SMD) for the association between PD and obesity.

### 2.8. Summary of Results

The main results of the included SRs were summarized, categorizing their findings into the following points: general association, by age, sex, countries, or continents, BMI, type of PD, smoking, and by periodontal clinical parameters (plaque index, gingival index, bleeding on probing, probing depth, and sub- and supragingival calculus).

## 3. Results

### 3.1. Review and Selection of Primary Studies

**Figure 1:**
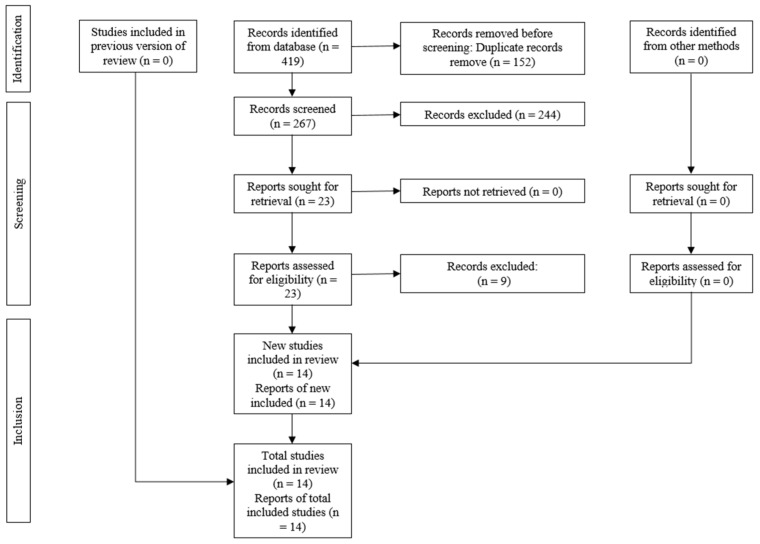
PRISMA diagram showing the process of inclusion and exclusion of studies.

The electronic database search retrieved 419 references, with 267 remaining after removal of duplicates. In phase 1, the title and abstract of the identified studies were assessed and 23 articles eligible for full-text reading were considered. Finally, 14 SRs remained for the qualitative synthesis. The reasons for the exclusion of the articles are shown in Table 2. The complete process of identification and selection of the studies is shown in Figure 1.

### 3.2. Review and Characteristics of Included Studies

The SRs included were published between 2010 and 2022. They were held in Brazil [42,43,44,45], South Korea [46], Qatar [47], Indonesia [48], Australia [49], Belgium [50], Spain [51], Denmark [52], China [53], United Kingdom [54], and United States [55]. Nine SRs [43,44,45,46,47,48,51,54,55] studied the association in adults, two in adolescents and adults [49,52], two in children and adolescents [50,53], and one in pregnant women [42]. More information on SR characteristics can be found in Table 3.

### 3.3. Assessment of Methodological Quality and Quality of Evidence

Nine SRs [42,43,44,46,49,52,53,54,55] were considered to have high confidence, four SRs [45,47,48,50] had low confidence, and one SR [51] had critically low confidence (Table 4).

### 3.4. Overlapping

A total of 397 primary studies were identified in the SRs. Of these, approximately 41.81% of the primary studies were included in more than one SR. Thirty studies were included twice; twenty-three were included three times; twelve were included four times; seven were included five times; four were included six times; and one was included seven times. More information on the overlap and characteristics of the primary studies is available in Table 5.

### 3.5. Synthesis of Results

The syntheses of the results are presented in Table 6.

#### 3.5.1. General Association

Nine SRs [42,45,46,47,48,49,50,51,55] included reported that there was an association between PD and obesity. Six SRs [42,45,46,48,50,55] meta-analyzed the results and found that the OR ranged from 1.23 (CI: 1.15 to 1.33) [48] to 1.46 (CI: 1.20 to 1.77) [50] and the RR was 2.21 (CI: 1.53 to 3.17) [42]. Abu-Shawish et al. [47] reported that the OR ranged from 1.77 to 3.25 and the RR ranged from 1.64 to 1.84, while Khan et al. [49] reported that the OR ranged between 1.1 and 4.5 and Martínez-Herrera et al. [51] reported that the OR ranged from 0.99 to 4.3, the HR ranged between 1.03 and 3.24, and the RR ranged from 0.99 to 5.4.

#### 3.5.2. Age

Three SRs [46,52,55] included reported that there was an association between PD and obesity according to age. Two SRs [46,55] meta-analyzed the results and found that the OR was 2.21 (CI: 1.26 to 3.89) [46], 1.53 (CI: 1.17 to 2.00) [46], 1.82 (CI: 1.16 to 2.83) [46], 1.35 (CI: 1.14 to 1.59) [55], and 1.21 (CI: 1.04 to 1.41) [55] for ages 18 to 34 years, 35 to 54 years, older and equal to 55 years, young, and old, respectively. Keller et al. [52] reported that the HR ranged from 1.30 to 3.24 and 1.09 to 1.70, the IRR was 1.3 and 1.2, and the PR was 1.01 and 0.99 for ages in obese and overweight people, respectively.

#### 3.5.3. Sex

One SR [55] included reported that there was an association between PD and obesity according to sex. This study meta-analyzed its results and found that the OR was 1.50 (CI: 1.27 to 1.77) for men and 1.75 (CI: 1.26 to 2.43) for women. 

#### 3.5.4. Country or Continent

Two SRs [46,55] included reported that there was an association between PD and obesity depending on the country or continent. All of them meta-analyzed the results and found that the OR for the United States, Brazil, Korea, and Japan ranged from 0.59 (CI: 0.19 to 1.65) [46] to 1.75 (CI: 1.48 to 2.06) [46], while the OR for European countries, East Asia, Europe and the Middle East, and other Asian countries ranged from 0.98 (CI: 0.49 to 1.95) [46] to 2.46 (CI: 1.11 to 5.46) [46]. 

#### 3.5.5. Obese

Three SRs [44,54,55] included reported that there was an association between PD and people with obesity, while one RS [43] reported that there was an association between gingivitis and obese people. They all meta-analyzed the results and found that the OR ranged from 1.52 (CI: 1.26 to 1.83) [55] to 1.81 (CI: 1.42 to 2.30) [54]. The SMD ranged from 0.05 (CI: −0.20 to 0.29) [43] to 1.10 (CI: 0.14 to 2.05) [43]. Furthermore, the RR was 1.34 (CI: 1.21 to 1.47) [44].

#### 3.5.6. Overweight

Three SRs [44,54,55] included reported that there was an association between PD and overweight people, while one RS [43] reported that this association did not exist. They all meta-analyzed the results and found that the OR ranged from 1.18 (CI: 1.00 to 1.39) [55] to 1.27 (CI: 1.06 to 1.51) [54]. The SMD ranged from 0.30 (CI: −0.03 to 0.62) [43] to 2.08 (CI: −0.60 to 4.77) [43]. Furthermore, the RR was 1.13 (CI: 1.06 to 1.20) [44]. 

#### 3.5.7. Smoker and Non-Smoker

One SR [55] included reported that there was an association between PD and obesity depending on whether the person did not smoke. This study meta-analyzed its results and found that the OR was 1.36 (CI: 0.98 to 1.88) for smokers and 2.08 (CI: 1.29 to 3.36) for non-smokers.

#### 3.5.8. Bleeding on Probing

One SR [53] included reported that there was an association between PD and obesity when the BOP was greater than 25%, while one RS [43] reported that there was such an association when the BOP was from people with gingivitis and who were overweight. They all meta-analyzed their results and found that the OR was 5.41 (CI: 2.75 to 10.63) [53], while the SMD for the obese ranged from 0.03 (CI: −0.23 to 0.28) [43] to 0.64 (CI: −0.37 to 1.65) [43] and, for those who were overweight, it ranged from 0.13 (CI: −0.04 to 0.30) [43] to 0.78 (CI: 0.52 to 1.03) [43].

#### 3.5.9. Gingival Index

One SR [43] included reported that there was an association between PD and overweight people according to their gingival index. This study meta-analyzed its results and found that the SMD for the obese ranged from 0.35 (CI: −0.21 to −0.91) to 2.13 (CI: −1.51 to 5.77) and, for overweight people, it ranged from 0.97 (CI: 0.45 to 1.49) to 3.52 (CI: 2.32 to 4.71).

#### 3.5.10. Plaque Index

One SR [53] included reported that there was an association between PD and obesity when the plaque index was greater than 25%. This study meta-analyzed its results and found that the OR was 4.75 (CI: 2.42 to 9.34).

#### 3.5.11. Probing Depth

One SR [53] included reported that there was an association between PD and obesity when probing depth was greater than 4 mm. This study meta-analyzed its results and found that the OR was 14.15 (CI: 5.10 to 39.25).

#### 3.5.12. Subgingival Calculus

One SR [53] included reported that there was an association between PD and obesity according to subgingival calculus. This study meta-analyzed its results and found that the OR was 3.07 (CI: 1.10 to 8.62).

#### 3.5.13. Supragingival Calculus

One SR [53] included reported that there was no association between PD and obesity according to supragingival calculus. This study meta-analyzed its results and found that the OR was 1.08 (CI: 0.60 to 1.94).

## 4. Discussion

In recent years, there has been increasing interest in evaluating and analyzing the relationship between PD and obesity. Numerous studies have investigated this topic and found evidence to support this association.

Currently, obesity and overweight are considered global health problems of epidemic proportions, classified as chronic inflammatory diseases by the National Institutes of Health (NIH) and the World Health Organization (WHO) [1]. The WHO has reported a significant increase in obesity rates worldwide in all age groups since 1975 [1]. Although initially attributed primarily to an energy imbalance between calories consumed and calories expended, it is now recognized that the causes of obesity and overweight are much more complex and involve environmental and genetic factors [1,130].

For more than 20 years, oral health researchers have investigated the possible relationship between obesity and PD. Several potential mechanisms linking the two conditions have been identified, including an exaggerated immune response in obese individuals [128,131], differences in the oral microbiome [132], and the release of proinflammatory cytokines by adipose tissue cells [133]. Other mechanisms include the role of several molecules, such as TNFα, leptin, and ghrelin, which are involved in inflammation and energy balance [130]. These findings support the possibility of a biological connection between PD and obesity.

An umbrella review in 2018 [130] that included 14 SRs on the relationship between periodontitis and obesity highlighted that obese people are more likely to suffer from periodontitis than those of normal weight. Furthermore, Khan et al. [49] also found a positive association between obesity and periodontitis in young adults and adolescents. These findings were supported by a longitudinal cohort study in Taiwan, that included more than 12,000 people and found a slightly increased risk of periodontitis in obese people, with an even higher risk in obese people over 65 years of age [134].

Previous studies of the relationship between periodontitis and obesity have been conducted primarily in animals or through cross-sectional, case–control, or cohort studies. Recently, however, intervention studies have recently emerged. For example, Suvan et al. [130] analyzed six SRs that included intervention studies, but the results were contradictory.

Most studies found no differences in gingival inflammation between obese and non-obese individuals, but higher levels were observed in obese people with periodontitis. In addition, there were variations in the measurement of obesity, with some studies using different measures such as waist–hip ratio (WHR) and waist circumference (WC), indicating the need for consistency in measurement tools in future studies [43]. It is also clear that there is a positive association between obesity and periodontitis at all age levels, although determination of a cause–effect relationship is premature at this time [1].

In the present study, a comprehensive literature search was conducted to summarize and analyze the available SRs on the association between PD and obesity, and 14 SRs were identified that met the selection criteria. Although SRs are a reliable source of scientific evidence, it is important to be cautious when interpreting their results due to the possibility of bias. The SRs included in this study showed certain limitations related to the selected primary studies: different types of study, different definition criteria for periodontal disease (gingivitis or periodontitis), and different population groups studied (children, adolescents, adults, and pregnant women). These limitations of the primary studies made it impossible to perform a meta-analysis.

Some studies included in the analysis had a high level of confidence, which could strengthen the evidence for the results and conclusions of the current study. However, the persistence of systematic reviews with lower confidence levels highlights the need for greater rigor in conducting research on this topic.

The assessment of the methodological quality of the included SRs was performed using the AMSTAR-2 tool, which is current and widely recognized. Some studies were found to have deficiencies in critical domains 7, 9, and 13 of this tool. These deficiencies included failure to provide a list of excluded studies with justification, inadequate use of techniques to assess risk of bias, and failure to consider such risk when interpreting or discussing results. These findings highlight the importance of addressing these elements in future SRs.

Furthermore, caution should be taken when interpreting the results of systematic reviews, as about 50% of the included primary studies are repeated in multiple reviews, which may lead to repeated re-evaluation of the same data. This may distort the perception of the amount of work conducted in the field. Although, it would be beneficial to conduct new SRs to address the methodological limitations recommended by Moher [135] due to the high degree of overlap between existing reviews.

### 4.1. Evidence Summary

In this umbrella review, we sought to clarify the association between PD and obesity through the collection and analysis of SRs and meta-analysis on this topic, identifying the following results:

The SRs included in this study suggest an overall positive and direct association between PD and obesity. This finding aligns with what was found by Suvan et al. [130] and Lavigne [1], who also reported on this association.

With regard to age, it was observed that the association between PD and obesity was stronger in young people. This may be due to the fact that young people today tend to adopt unhealthy eating habits, which may contribute to both obesity and oral health problems [136,137].

Regarding gender, it was observed that this association was more present in women. This may be because hormonal changes during the menstrual cycle, pregnancy, and menopause affect fat metabolism in women, generally resulting in a higher percentage of body fat in women compared to men [130,138,139,140].

In relation to the country or continent, it was observed that the association was present in most countries and continents. This may be attributed to globalization and the adoption of Western lifestyles in many countries, which has led to the increase in unhealthy eating habits, smoking, and lack of physical activity [130,141].

In obese and overweight individuals, this association was found to be more pronounced in obese individuals. This may be due to unhealthy eating habits and lack of physical activity in this population group [130].

In relation to smoking, the association is more present in non-smokers. This may be explained by the tendency of smokers to have a weakened immune response and a greater propensity to inflammation, which could obscure this association. On the other hand, non-smokers may have a more pronounced inflammatory response to the inflammatory effects of obesity [52].

Regarding periodontal clinical indicators, the association was observed in all clinical aspects. This could be due to the fact that obesity is linked to modifications in the immune response and systemic inflammation, which negatively impacts periodontal health [1].

### 4.2. Implications for Clinical Practice

Oral health professionals have a responsibility to raise awareness and educate patients about how overweight and obesity can increase the risk of developing PD. Promoting good oral hygiene, including regular tooth brushing, flossing, and mouthwash, can help prevent plaque buildup and reduce the risk of PD. In the era of personalized medicine, it is suggested to incorporate BMI measurement as part of routine risk assessment and educate patients about the complex, multiorgan nature of obesity. It is crucial to implement preventive interventions to modify risk factors such as diet, exercise, and weight control, which may decrease the likelihood of obesity and PD. Additionally, a monitoring and follow-up plan should be established for patients with obesity, including frequent visits to the dentist and specific evaluations to detect PD early and provide intervention when necessary. Collaboration with endocrinologists, nutritionists and other specialists is essential for a comprehensive approach to the management of patients with obesity, allowing for co-ordinated medical and dental care.

### 4.3. Implications for Research

This review highlights the need to improve the presentation of SRs. The authors suggest the use of quality assessment tools to guide the development of future SRs. They also emphasize the importance of conducting primary studies with high methodological rigor to obtain more reliable results.

For future research on this topic, it is recommended to standardize the diagnostic criteria for both PD and obesity, conduct high-quality prospective studies with larger samples and consistent measures, and conduct more robust research to understand the precise mechanisms and the magnitude of the association between PD and obesity.

## 5. Conclusions

Based on the results and conclusions of the SRs with a high overall confidence, PD is associated with obesity in children, adolescents, adults, and pregnant women.

## Figures and Tables

**Table 1:** Database search strategy.

Database	Search Strategy	Number of Studies
Pubmed	((“periodontitis”) OR (“periodontal disease”) OR (“furcation defect”) OR (“gingival disease”) OR (“gingivitis”) OR (“tooth loss”) OR (“tooth migration”) OR (“tooth mobility”) OR (“missing teeth”) OR (“periodontal inflammation”) OR (“gum disease”) OR (“gum inflammation”)) AND ((“Obesity”) OR (“overweight”) OR (“body weight”) OR (“body mass index”) OR (“abdominal fat”) OR (“obese”) OR (“BMI”)) AND ((“systematic review”) OR (“meta-analysis”))	76
Cochrane database	#1 MeSH descriptor: [Periodontal Diseases] explode all trees	42
	#2 MeSH descriptor: [Periodontitis] in all MeSH products	
	#3 MeSH descriptor: [Furcation Defects] explode all trees	
	#4 MeSH descriptor: [Gingival Diseases] explode all trees	
	#5 MeSH descriptor: [Gingivitis] explode all trees	
	#6 MeSH descriptor: [Tooth Loss] explode all trees	
	#7 MeSH descriptor: [Tooth Migration] explode all trees	
	#8 MeSH descriptor: [Tooth Mobility] explode all trees	
	#9 (“periodontitis”) OR (“periodontal disease”) OR (“furcation defect”) OR (“gingival disease”) OR (“gingivitis”) OR (“tooth loss”) OR (“tooth migration”) OR (“tooth mobility”) OR (“missing teeth”) OR (“periodontal inflammation”) OR (“gum disease”) OR (“gum inflammation”) (Word variations have been searched)	
	#10 #1 OR #2 OR #3 OR #4 OR #5 OR #6 OR #7 OR #8 OR #9	
	#11 MeSH descriptor: [Obesity] explode all trees	
	#12 MeSH descriptor: [Overweight] explode all trees	
	#13 MeSH descriptor: [Body Weight] explode all trees	
	#14 MeSH descriptor: [Body Mass Index] explode all trees	
	#15 MeSH descriptor: [Abdominal Fat] explode all trees	
	#16 (“obesity”) OR (“overweight”) OR (“body weight”) OR (“body mass index”) OR (“abdominal fat”) (Word variations have been searched) OR (“obese”) OR (“BMI”) (Word variations have been searched)	
	#17 #11 OR #12 OR #13 OR #14 OR #15 OR #16	
	#18 MeSH descriptor: [Systematic Reviews as Topic] explode all trees	
	#19 MeSH descriptor: [Meta-Analysis as Topic] explode all trees	
	#20 (“systematic review”) OR (“meta-analysis”) (Word variations have been searched)	
	#21 #18 OR #19 OR #20; #22 #10 AND #17 AND #21	
Scielo	((((“periodontitis”) OR (“periodontal disease”) OR (“furcation defect”) OR (“gingival disease”) OR (“gingivitis”) OR (“tooth loss”) OR (“tooth migration”) OR (“tooth mobility”) OR (“missing teeth”) OR (“periodontal inflammation”) OR (“gum disease”) OR (“gum inflammation”))) AND (((“Obesity”) OR (“overweight”) OR (“body weight”) OR (“body mass index”) OR (“abdominal fat”) OR (“obese”) OR (“BMI”)))) AND (((“systematic review”) OR (“meta-analysis”)))	3
Scopus	(TITLE-ABS-KEY (((“periodontitis”) OR (“periodontal disease”) OR (“furcation defect”) OR (“gingival disease”) OR (“gingivitis”) OR (“tooth loss”) OR (“tooth migration”) OR (“tooth mobility”) OR (“missing teeth”) OR (“periodontal inflammation”) OR (“gum disease”) OR (“gum inflammation”))) AND TITLE-ABS-KEY (((“Obesity”) OR (“overweight”) OR (“body weight”) OR (“body mass index”) OR (“abdominal fat”) OR (“obese”) OR (“BMI”))) AND TITLE-ABS-KEY (((“systematic review”) OR (“meta-analysis”)))) AND (LIMIT-TO (DOCTYPE, “re”) OR LIMIT-TO (DOCTYPE, “ar”)) AND (LIMIT-TO (PUBSTAGE, “final”)) AND (LIMIT-TO (SRCTYPE, “j”))	174
Web of Science	(TS=(“periodontitis”) OR TS=(“periodontal disease”) OR TS=(“furcation defect”) OR TS=(“gingival disease”) OR TS=(“gingivitis”) OR TS=(“tooth loss”) OR TS=(“tooth migration”) OR TS=(“tooth mobility”) OR TS=(“missing teeth”) OR TS=(“periodontal inflammation”) OR TS=(“gum disease”) OR TS=(“gum inflammation”)) AND (TS=(“obesity”) OR TS=(“overweight”) OR TS=(“body weight”) OR TS=(“body mass index”) OR TS=(“abdominal fat”) OR TS=(“obese”) OR TS=(“BMI”)) AND (TS=(“systematic review”) OR TS=(“meta-analysis”))	79
Google Scholar	allintitle: ((“periodontal disease”) OR (“periodontitis”)) + ((“Obesity”) OR (“overweight”)) + ((“systematic review”) OR (“meta-analysis”))	22
Proquest Dissertations and Theses	((“periodontal disease”) OR (“periodontitis”)) AND ((“Obesity”) OR (“overweight”)) AND ((“systematic review”) OR (“meta-analysis”)) AND NOT ((“cardiovascular”) OR (“obstructive sleep apnea”) OR (“cancer”) OR (“pregnant”) OR (“dementia”) OR (“in vitro”) OR (“dental caries”) OR (“fractures”) OR (“rat”) OR (“diabetes mellitus”) OR (“periodontal treatment”) OR (“pulpotomy”) OR (“treatment”))	23
OpenGrey	((“periodontitis”) OR (“periodontal disease”) OR (“furcation defect”) OR (“gingival disease”) OR (“gingivitis”) OR (“tooth loss”) OR (“tooth migration”) OR (“tooth mobility”) OR (“missing teeth”) OR (“periodontal inflammation”) OR (“gum disease”) OR (“gum inflammation”)) AND ((“Obesity”) OR (“overweight”) OR (“body weight”) OR (“body mass index”) OR (“abdominal fat”) OR (“obese”) OR (“BMI”)) AND ((“systematic review”) OR (“meta-analysis”))	0

**Table 2:** Reason for exclusion of studies.

Author	Reason for Exclusion
Paranhos et al. [33]	They associated obesity with periodontal treatment
Zhang et al. [34]	
Joseph et al. [35]	
Akram et al. [36]	
Nascimento et al. [37]	
Gerber et al. [38]	
Papageorgiou et al. [39]	
Deng et al. [40]	They associated obesity with salivary biomarkers of PD
Akram et al. [41]	

**Table 3:** Characteristics of included studies.

Authors	Year	Study Design	Country	Included Study Design	Number of Studies in the Qualitative Analysis	Number of Studies in the Quantitative Analysis	Outcomes		Conclusions
Foratori-Junior et al. [42]	2022	SR with MA	Brazil	CS, C, and CC	11	11	General	RR = 2.21 (1.53–3.17)	There is an association between overweight/obesity and periodontitis during pregnancy.
Kim et al. [46]	2022	SR with MA	South Korea	CS, C, and CC	37	29	General	OR = 1.35 (1.05–1.75)	A positive association was found between obesity and periodontitis regardless of country or age.
							18–34 years	OR = 2.21 (1.26–3.89)	
							35–54 years	OR = 1.53 (1.17–2.00)	
							≥ 55 years	OR = 1.82 (1.16–2.83)	
							United States	OR = 0.59 (0.19–1.65)	
							Brazil	OR = 1.70 (0.78–3.72)	
							European countries	OR = 2.46 (1.11–5.46)	
							Korea	OR = 1.34 (1.00–1.80)	
							Japan	OR = 1.75 (1.48–2.06)	
							Other Asian countries	OR = 0.98 (0.49–1.95)	
Abu-Shawish et al. [47]	2022	SR	Qatar	CS, C, and CC	15	0	General	OR = 1.77–3.25	This SR found a positive association between obesity in terms of increased BMI and periodontitis in adults.
								RR = 1.64–1.84	
Khairunnisa et al. [48]	2021	SR with MA	Indonesia	CS	11	11	General	OR = 1.23 (1.15–1.33)	Obesity increases periodontitis in adults.
da Silva et al. [43]	2021	SR with MA	Brazil	CS, CT, C, and CC	90	90	Obese	SMD = 0.05 (−0.20–0.29)	Higher measures of gingival inflammation can be expected for those with higher BMI.
							Overweight	SMD = 0.30 (−0.03–0.62)	
							Overweight or obese	SMD = 0.20 (−0.09–0.48)	
							BOP (obese)	SMD = 0.03 (−0.23–0.28)	
							BOP (Overweight)	SMD = 0.13 (−0.04–0.30)	
							BOP (Overweight or obese)	SMD = 0.20 (−0.05–0.45)	
							GI (obese)	SMD = 0.35 (−0.21–0.91)	
							GI (Overweigh)	SMD = 0.97 (0.45–1.49)	
							GI (Overweight or obese)	SMD = 0.22 (−0.24–0.68)	
							Obese—G	SMD = 1.10 (0.14–2.05)	
							Overweight—G	SMD = 2.08 (−0.60–4.77)	
							Overweight or obese—G	SMD = 2.91 (−0.89–6.72)	
							BOP (obese)—G	SMD = 0.64 (−0.37–1.65)	
							BOP (Overweight)—G	SMD = 0.78 (0.52–1.03)	
							BOP (Overweight or obese)—G	SMD = 1.02 (0.77–1.27)	
							GI (obese)—G	SMD = 2.13 (−1.51–5.77)	
							GI (Overweight)—G	SMD = 3.52 (2.32–4.71)	
							GI (Overweight or obese)—G	SMD = 4.91 (3.64–6.17)	
Khan et al. [49]	2018	SR	Australia	CS, C, and CC	25	0	General	OR = 1.1–4.5	There was evidence to suggest that obesity is associated with periodontitis in adolescents and young adults.
Martens et al. [50]	2017	SR with MA	Belgium	CS, C, and CC	12	7	General	OR = 1.46 (1.20–1.77)	The available evidence suggests a significantly positive association between periodontal disease and obesity in children.
Martinez-Herrera et al. [51]	2017	SR	Spain	C, CC, and CT	28	0	General	OR = 0.99–4.3	The association between obesity and periodontitis was consistent with a compelling pattern of increased risk of periodontitis in overweight or obese individuals.
								HR = 1.03–3.24	
								RR = 0.99–5.4	
Nascimento et al. [44]	2015	SR with MA	Brazil	O	5	5	Overweight	RR = 1.13 (1.06–1.20)	A clear positive association between weight gain and new cases of periodontitis was found.
							Obese	RR = 1.34 (1.21–1.47)	
Keller et al. [52]	2015	SR	Denmark	C and CT	13	0	Age (obese)	HR = 1.30–3.24	Overweight and obesity can be risk factors for the development or worsening of periodontal health.
								IRR = 1.3	
								PR = 1.01	
							Age (overweight)	HR = 1.09–1.70	
								IRR = 1.2	
								PR = 0.99	
Li et al. [53]	2015	SR with MA	China	CS and CC	16	5	PI > 25%	OR = 4.75 (2.42–9.34)	Obesity is associated with some signs of periodontal disease in children and adolescents.
							BOP > 25%	OR = 5.41 (2.75–10.63)	
							SBC	OR = 3.07 (1.10–8.62)	
							SPC	OR = 1.08 (0.60–1.94)	
							PD > 4 mm	OR = 14.15 (5.10–39.25)	
de Moura-Grec et al. [45]	2014	SR with MA	Brazil	CS	31	31	General	OR = 1.30 (1.25–1.35)	Obesity was associated with periodontitis; however, the risk factors that aggravate these diseases should be better clarified to elucidate the direction of this association.
Suvan et al. [54]	2011	SR with MA	United Kingdom	CS, C, and CC	33	19	Obese	OR = 1.81 (1.42–2.30)	These results support an association between BMI overweight and obesity and periodontitis although the magnitude is unclear.
							Overweight	OR = 1.27 (1.06–1.51)	
							Overweight and obese	OR = 2.13 (1.40–3.26)	
Chaffee et al. [55]	2010	SR with MA	United States	O	70	28	General	OR = 1.35 (1.23–1.47)	There is a positive association between periodontal disease and obesity, which was consistent and coherent with a biologically plausible role of obesity in the development of periodontal disease.
							Obese	OR = 1.52 (1.26–1.83)	
							Overweight	OR = 1.18 (1.00–1.39)	
							East Asia	OR = 1.32 (1.19–1.47)	
							Europe and Middle East	OR = 1.87 (1.17–2.99)	
							United States	OR = 1.30 (1.16–1.46)	
							Men	OR = 1.50 (1.27–1.77)	
							Women	OR = 1.75 (1.26–2.43)	
							Young	OR = 1.35 (1.14–1.59)	
							Older	OR = 1.21 (1.04–1.41)	
							Smoker	OR = 1.36 (0.98–1.88)	
							Non-smoker	OR = 2.08 (1.29–3.36)	

SR = systematic review; MA = meta-analysis; O = observational study; CT = clinical trial; CS = cross-sectional; C = cohort; CC = case and control; BMI = body mass index; G = gingivitis; BOP = bleeding on probing; PD = probing depth; PI = plaque index; GI = gingival index; SBC = subgingival calculus; SPC = supragingival calculus; OR = odds ratio; RR = risk/rate ratio; HR = hazard ratio; PR = prevalence ratio; IRR = incidence risk ratio.

**Table 4:** Assessment of the methodological quality and the quality of the evidence of the included studies.

Authors	Year	AMSTAR-2																Overall Confidence
		1	2 *	3	4 *	5	6	7 *	8	9 *	10	11 *	12	13 *	14	15 *	16	
Foratori-Junior et al. [42]	2022	Yes	Yes	Yes	Yes	Yes	Yes	Yes	Yes	Yes	Yes	Yes	Yes	Yes	Yes	Yes	Yes	High
Kim et al. [46]	2022	Yes	Yes	Yes	Yes partial	Yes	Yes	Yes partial	Yes	Yes	Yes	Yes	Yes	Yes	Yes	Yes	Yes	High
Abu-Shawish et al. [47]	2022	Yes	Yes	Yes	Yes partial	Yes	Yes	No	Yes	Yes	Yes	No meta-analysis		Yes	Yes	No meta-analysis	Yes	Low
Khairunnisa et al. [48]	2021	Yes	Yes partial	Yes	Yes partial	No	No	Yes partial	No	Yes partial	Yes	Yes	No	No	No	Yes	Yes	Low
da Silva et al. [43]	2021	Yes	Yes partial	Yes	Yes partial	Yes	Yes	Yes	Yes	Yes	Yes	Yes	Yes	Yes	Yes	Yes	Yes	High
Khan et al. [49]	2018	Yes	Yes	Yes	Yes partial	Yes	Yes	Yes partial	Yes	Yes	Yes	No meta-analysis		Yes	Yes	No meta-analysis	Yes	High
Martens et al. [50]	2017	Yes	Yes partial	Yes	Yes	Yes	Yes	No	Yes	Yes partial	Yes	Yes	Yes	Yes	Yes	Yes	Yes	Low
Martinez-Herrera et al. [51]	2017	Yes	Yes partial	Yes	Yes partial	No	No	No	Yes	No	No	No meta-analysis		No	No	No meta-analysis	Yes	Critically low
Nascimento et al. [44]	2015	Yes	Yes partial	Yes	Yes partial	Yes	Yes	Yes	Yes	Yes	Yes	Yes	Yes	Yes	Yes	Yes	Yes	High
Keller et al. [52]	2015	Yes	Yes partial	Yes	Yes partial	Yes	Yes	Yes	Yes	Yes partial	Yes	No meta-analysis		Yes	Yes	No meta-analysis	Yes	High
Li et al. [53]	2015	Yes	Yes partial	Yes	Yes partial	Yes	Yes	Yes partial	Yes	Yes partial	Yes	Yes	Yes	Yes	Yes	Yes	Yes	High
de Moura-Grec et al. [45]	2014	Yes	Yes partial	Yes	Yes partial	Yes	Yes	Yes partial	Yes	No	Yes	Yes	Yes	Yes	Yes	Yes	No	Low
Suvan et al. [54]	2011	Yes	Yes partial	Yes	Yes	Yes	Yes	Yes partial	Yes	Yes	Yes	Yes	Yes	Yes	Yes	Yes	Yes	High
Chaffee et al. [55]	2010	Yes	Yes partial	Yes	Yes	Yes	Yes	Yes partial	Yes	Yes partial	Yes	Yes	Yes	Yes	Yes	Yes	Yes	High

AMSTAR = A MeaSurement Tool to Assess Systemic Reviews. 1 = Did the research questions and inclusion criteria for the review include the components of PICO? 2 = Did the report of the review contain an explicit statement that the review methods were established prior to the conduct of the review and did the report justify any significant deviations from the protocol? 3 = Did the review authors explain their selection of the study designs for inclusion in the review? 4 = Did the review authors use a comprehensive literature search strategy? 5 = Did the review authors perform study selection in duplicate? 6 = Did the review authors perform data extraction in duplicate? 7 = Did the review authors provide a list of excluded studies and justify the exclusions? 8 = Did the review authors describe the included studies in adequate detail? 9 = Did the review authors use a satisfactory technique for assessing the risk of bias (RoB) in individual studies that were included in the review? 10 = Did the review authors report on the sources of funding for the studies included in the review? 11 = If meta-analysis was performed, did the review authors use appropriate methods for statistical combination of results? 12 = If meta-analysis was performed, did the review authors assess the potential impact of RoB in individual studies on the results of the meta-analysis or other evidence synthesis? 13 = Did the review authors account for RoB in primary studies when interpreting/discussing the results of the review? 14 = Did the review authors provide a satisfactory explanation for, and discussion of, any heterogeneity observed in the results of the review? 15 = If they performed quantitative synthesis, did the review authors carry out an adequate investigation of publication bias (small study bias) and discuss its likely impact on the results of the review? 16 = Did the review authors report any potential sources of conflict of interest, including any funding they received for conducting the review? * = Critical domain.

**Table 5:** Overlapping of primary studies in systematic reviews.

Primary Studies	Systematic Reviews That Included the Primary Studies	Times That Primary Studies Were Included
Khader et al. [56]	Kim et al. [46], Abu-Shawish et al. [47], Khairunnisa et al. [48], da Silva et al. [43], de Moura-Grec et al. [45], Suvan et al. [54], Chaffee et al. [55]	7
Kongstad et al. [57]	Kim et al. [46], Khairunnisa et al. [48], Martínez-Herrera et al. [51], de Moura-Grec et al. [45], Suvan et al. [54], Chaffee et al. [55]	6
Ekuni et al. [58]	Kim et al. [46], Khairunnisa et al. [48], Khan et al. [49], de Moura-Grec et al. [45], Suvan et al. [54], Chaffee et al. [55]	6
Dalla Vecchia et al. [59]	Kim et al. [46], Abu-Shawish et al. [47], Khairunnisa et al. [48], de Moura-Grec et al. [45], Suvan et al. [54], Chaffee et al. [55]	6
Al-Zahrani et al. [60]	Kim et al. [46], Khan et al. [49], Martínez-Herrera et al. [51], de Moura-Grec et al. [45], Suvan et al. [54], Chaffee et al. [55]	6
Pataro et al. [61]	Kim et al. [46], Abu-Shawish et al. [47], Khairunnisa et al. [48], da Silva et al. [43], Martínez-Herrera et al. [51]	5
Han et al. [62]	Kim et al. [46], Khairunnisa et al. [48], de Moura-Grec et al. [45], Suvan et al. [54], Chaffee et al. [55]	5
Saxlin et al. [63]	Kim et al. [46], Martínez-Herrera et al. [51], Nascimento et al. [44], Keller et al. [52], de Moura-Grec et al. [45]	5
Haffajee et al. [64]	Kim et al. [46], da Silva et al. [43], de Moura-Grec et al. [45], Suvan et al. [54], Chaffee et al. [55]	5
Linden et al. [65]	Abu-Shawish et al. [47], Martínez-Herrera et al. [51], Keller et al. [52], Suvan et al. [54], Chaffee et al. [55]	5
Wood et al. [28]	Khan et al. [49], Martínez-Herrera et al. [51], de Moura-Grec et al. [45], Suvan et al. [54], Chaffee et al. [55]	5
Saito et al. [66]	Kim et al. [46], Martínez-Herrera et al. [51], de Moura-Grec et al. [45], Suvan et al. [54], Chaffee et al. [55]	5
Ekuni et al. [67]	da Silva et al. [43], Martínez-Herrera et al. [51], Nascimento et al. [44], Keller et al. [52]	4
Amin et al. [68]	Abu-Shawish et al. [47], da Silva et al. [43], Khan et al. [49], Chaffee et al. [55]	4
Dumitrescu et al. [69]	Kim et al. [46], da Silva et al. [43], de Moura-Grec et al. [45], Chaffee et al. [55]	4
Furuta et al. [70]	Kim et al. [46], Khan et al. [49], de Moura-Grec et al. [45], Chaffee et al. [55]	4
Kumar et al. [71]	Kim et al. [46], Khairunnisa et al. [48], de Moura-Grec et al. [45], Chaffee et al. [55]	4
Kushiyama et al. [72]	Kim et al. [46], de Moura-Grec et al. [45], Suvan et al. [54], Chaffee et al. [55]	4
Sarlati et al. [73]	Abu-Shawish et al. [47], Khan et al. [49], Suvan et al. [54], Chaffee et al. [55]	4
Borges-Yañez et al. [74]	Kim et al. [46], de Moura-Grec et al. [45], Suvan et al. [54], Chaffee et al. [55]	4
Reeves et al. [75]	Khan et al. [49], Martens et al. [50], Li et al. [53], Chaffee et al. [55]	4
Genco et al. [76]	Martínez-Herrera et al. [51], de Moura-Grec et al. [45], Suvan et al. [54], Chaffee et al. [55]	4
Saito et al. [77]	Martínez-Herrera et al. [51], de Moura-Grec et al. [45], Suvan et al. [54], Chaffee et al. [55]	4
Torrungruang et al. [78]	Kim et al. [46], de Moura-Grec et al. [45], Suvan et al. [54], Chaffee et al. [55]	4
Gulati et al. [79]	Kim et al. [46], Khairunnisa et al. [48], da Silva et al. [43]	3
Buduneli et al. [80]	Abu-Shawish et al. [47], da Silva et al. [43], Martínez-Herrera et al. [51]	3
Fadel et al. [81]	Khan et al. [49], Martens et al. [50], Li et al. [53]	3
Suvan et al. [22]	da Silva et al. [43], Martínez-Herrera et al. [51], Keller et al. [52]	3
Altay et al. [82]	da Silva et al. [43], Martínez-Herrera et al. [51], Keller et al. [52]	3
Irigoyen-Camacho et al. [83]	Khan et al. [49], Martens et al. [50], Li et al. [53]	3
Al-Zahrani et al. [84]	da Silva et al. [43], Martínez-Herrera et al. [51], Keller et al. [52]	3
de Castilhos et al. [85]	da Silva et al. [43], Khan et al. [49], Keller et al. [52]	3
Gorman et al. [86]	Martínez-Herrera et al. [51], Nascimento et al. [44], Keller et al. [52]	3
Jimenez et al. [87]	Martínez-Herrera et al. [51], Nascimento et al. [44], Keller et al. [52]	3
Zeigler et al. [88]	Khan et al. [49], Martens et al. [50], Li et al. [53]	3
Franchini et al. [89]	Khan et al. [49], Martens et al. [50], Li et al. [53]	3
Modéer et al. [90]	Khan et al. [49], Martens et al. [50], Li et al. [53]	3
Morita et al. [91]	Martínez-Herrera et al. [51], Nascimento et al. [44], Keller et al. [52]	3
Zuza et al. [92]	da Silva et al. [43], Martínez-Herrera et al. [51], Keller et al. [52]	3
Morita et al. [93]	Kim et al. [46], de Moura-Grec et al. [45], Chaffee et al. [55]	3
Saxlin et al. [94]	Kim et al. [46], de Moura-Grec et al. [45], Chaffee et al. [55]	3
Wang et al. [95]	Martínez-Herrera et al. [51], Suvan et al. [54], Chaffee et al. [55]	3
D’aiuto et al. [96]	Kim et al. [46], de Moura-Grec et al. [45], Chaffee et al. [55]	3
Saxlin et al. [97]	Martínez-Herrera et al. [51], de Moura-Grec et al. [45], Chaffee et al. [55]	3
Ylöstalo et al. [98]	Martínez-Herrera et al. [51], Suvan et al. [54], Chaffee et al. [55]	3
Shimazaki et al. [99]	de Moura-Grec et al. [45], Suvan et al. [54], Chaffee et al. [55]	3
Machado et al. [100]	Kim et al. [46], de Moura-Grec et al. [45], Chaffee et al. [55]	3
Caracho et al. [101]	Foratori-Junior et al. [42], da Silva et al. [43]	2
Foratori-Junior et al. [102]	Foratori-Junior et al. [42], da Silva et al. [43]	2
Fusco et al. [103]	Foratori-Junior et al. [42], da Silva et al. [43]	2
Deshpande et al. [19]	Abu-Shawish et al. [47], da Silva et al. [43]	2
Kim et al. [104]	Kim et al. [46], Khairunnisa et al. [48]	2
Martínez-Herrera et al. [105]	Kim et al. [46], da Silva et al. [43]	2
Nascimento et al. [106]	Abu-Shawish et al. [47], da Silva et al. [43]	2
Al Habashneh et al. [107]	Kim et al. [46], da Silva et al. [43]	2
Balli et al. [108]	da Silva et al. [43], Martínez-Herrera et al. [51]	2
Öngöz Dede et al. [109]	da Silva et al. [43], Martínez-Herrera et al. [51]	2
Bouaziz et al. [110]	da Silva et al. [43], Martínez-Herrera et al. [51]	2
Gonçalves et al. [111]	da Silva et al. [43], Martínez-Herrera et al. [51]	2
Peng et al. [112]	Martens et al. [50], Li et al. [53]	2
Nascimento et al. [113]	Martens et al. [50], Li et al. [53]	2
Scorzetti et al. [114]	Martens et al. [50], Li et al. [53]	2
Benguigui et al. [115]	Kim et al. [46], da Silva et al. [43]	2
Kim et al. [116]	Khairunnisa et al. [48], Martínez-Herrera et al. [51]	2
Modéer et al. [117]	Martens et al. [50], Li et al. [53]	2
Shimazaki et al. [118]	Kim et al. [46], Chaffee et al. [55]	2
Li et al. [119]	Kim et al. [46], de Moura-Grec et al. [45]	2
Pitiphat et al. [120]	de Moura-Grec et al. [45], Chaffee et al. [55]	2
Saito [121]	Kim et al. [46], de Moura-Grec et al. [45]	2
Saito et al. [122]	Suvan et al. [54], Chaffee et al. [55]	2
Wood et al. [123]	de Moura-Grec et al. [45], Chaffee et al. [55]	2
Alabdulkarin et al. [124]	Suvan et al. [54], Chaffee et al. [55]	2
Chapper et al. [125]	Suvan et al. [54], Chaffee et al. [55]	2
Nishida et al. [126]	Suvan et al. [54], Chaffee et al. [55]	2
Socransky et al. [127]	Suvan et al. [54], Chaffee et al. [55]	2
Lundin et al. [128]	Khan et al. [49], de Moura-Grec et al. [45]	2
Buhlin et al. [129]	Suvan et al. [54], Chaffee et al. [55]	2

**Table 6:** Synthesis of the results of the included studies.

Authors	Outcome		Association
Foratori-Junior et al. [42]	General	RR = 2.21 (1.53–3.17)	Yes
Kim et al. [46]	General	OR = 2.21 (1.26–3.89)	Yes
	18–34 years	OR = 1.35 (1.05–1.75)	Yes
	35–54 years	OR = 1.53 (1.17–2.00)	Yes
	≥55 years	OR = 1.82 (1.16–2.83)	Yes
	United States	OR = 0.59 (0.19–1.65)	No
	Brazil	OR = 1.70 (0.78–3.72)	No
	European countries	OR = 2.46 (1.11–5.46)	Yes
	Korea	OR = 1.34 (1.00–1.80)	Yes
	Japan	OR = 1.75 (1.48–2.06)	Yes
	Other Asian countries	OR = 0.98 (0.49–1.95)	No
Abu-Shawish et al. [47]	General	OR = 1.77–3.25	Yes
		RR = 1.64–1.84	Yes
Khairunnisa et al. [48]	General	OR = 1.23 (1.15–1.33)	Yes
da Silva et al. [43]	Obese	SMD = 0.05 (−0.20–0.29)	No
	Overweight	SMD = 0.30 (−0.03–0.62)	No
	Overweight or obese	SMD = 0.20 (−0.09–0.48)	No
	BOP (obese)	SMD = 0.03 (−0.23–0.28)	No
	BOP (Overweight)	SMD = 0.13 (−0.04–0.30)	No
	BOP (Overweight or obese)	SMD = 0.20 (−0.05–0.45)	No
	GI (obese)	SMD = 0.35 (−0.21–0.91)	No
	GI (Overweight)	SMD = 0.97 (0.45–1.49)	Yes
	GI (Overweight or obese)	SMD = 0.22 (−0.24–0.68)	No
	Obese—G	SMD = 1.10 (0.14–2.05)	Yes
	Overweight—G	SMD = 2.08 (−0.60–4.77)	No
	Overweight or obese—G	SMD = 2.91 (−0.89–6.72)	No
	BOP (obese)—G	SMD = 0.64 (−0.37–1.65)	No
	BOP (Overweight)—G	SMD = 0.78 (0.52–1.03)	Yes
	BOP (Overweight or obese)—G	SMD = 1.02 (0.77–1.27)	Yes
	GI (obese)—G	SMD = 2.13 (−1.51–5.77)	No
	GI (Overweight)—G	SMD = 3.52 (2.32–4.71)	Yes
	GI (Overweight or obese)—G	SMD = 4.91 (3.64–6.17)	Yes
Khan et al. [49]	General	OR = 1.1–4.5	Yes
Martens et al. [50]	General	OR = 1.46 (1.20–1.77)	Yes
Martinez-Herrera et al. [51]	General	OR = 0.99–4.3	Yes
		HR = 1.03–3.24	Yes
		RR = 0.99–5.4	Yes
Nascimento et al. [44]	Overweight	RR = 1.13 (1.06–1.20)	Yes
	Obese	RR = 1.34 (1.21–1.47)	Yes
Keller et al. [52]	Age (obese)	HR = 1.30–3.24	Yes
		IRR = 1.3	Yes
		PR = 1.01	Yes
	Age (overweight)	HR = 1.09–1.70	Yes
		IRR = 1.2	Yes
		PR = 0.99	Yes
Li et al. [53]	PI > 25%	OR = 4.75 (2.42–9.34)	Yes
	BOP > 25%	OR = 5.41 (2.75–10.63)	Yes
	SBC	OR = 3.07 (1.10–8.62)	Yes
	SPC	OR = 1.08 (0.60–1.94)	No
	PD > 4 mm	OR = 14.15 (5.10–39.25)	Yes
de Moura-Grec et al. [45]	General	OR = 1.30 (1.25–1.35)	Yes
Suvan et al. [54]	Obese	OR = 1.30 (1.25–1.35)	Yes
	Overweight	OR = 1.81 (1.42–2.30)	Yes
	Overweight and obese	OR = 1.27 (1.06–1.51)	Yes
Chaffee et al. [55]	General	OR = 1.35 (1.23–1.47)	Yes
	Obese	OR = 1.52 (1.26–1 83)	Yes
	Overweight	OR = 1.18 (1.00–1.39)	Yes
	East Asia	OR = 1.32 (1.19–1.47)	Yes
	Europe and Middle East	OR = 1.87 (1.17–2.99)	Yes
	United States	OR = 1.30 (1.16–1.46)	Yes
	Men	OR = 1.50 (1.27–1.77)	Yes
	Women	OR = 1.75 (1.26–2.43)	Yes
	Young	OR = 1.35 (1.14–1.59)	Yes
	Older	OR = 1.21 (1.04–1.41)	Yes
	Smoker	OR = 1.36 (0.98–1.88)	No
	Non-smoker	OR = 2.08 (1.29–3.36)	Yes

G = gingivitis; BOP = bleeding on probing; PD = probing depth; PI = plaque index; GI = gingival index; SBC = subgingival calculus; SPC = supragingival calculus; OR = odds ratio; RR = risk/rate ratio; HR = hazard ratio; PR = prevalence ratio; IRR = incidence risk ratio.
